# Reading and conducting instrumental variable studies: guide, glossary, and checklist

**DOI:** 10.1136/bmj-2023-078093

**Published:** 2024-10-14

**Authors:** Venexia Walker, Eleanor Sanderson, Michael G Levin, Scott M Damraurer, Timothy Feeney, Neil M Davies

**Affiliations:** 1 Medical Research Council Integrative Epidemiology Unit at the University of Bristol, Bristol, UK; 2 Population Health Sciences, Bristol Medical School, University of Bristol, Bristol, UK; 3 Division of Cardiovascular Medicine, Department of Medicine, University of Pennsylvania Perelman School of Medicine, Philadelphia, PA, USA; 4 Department of Surgery and Department of Genetics, Perelman School of Medicine, University of Pennsylvania, Philadelphia, PA, USA; 5 Corporal Michael J Crescenz VA Medical Center, Philadelphia, PA, USA; 6 Department of Epidemiology, University of North Carolina Chapel Hill, Chapel Hill, NC, USA; 7 Division of Psychiatry, University College London, London W1T 7NF, UK; 8 Department of Statistical Science, University College London, London, UK; 9 K G Jebsen Centre for Genetic Epidemiology, Department of Public Health and Nursing, Norwegian University of Science and Technology, Trondheim, Norway

## Abstract

Instrumental variable analysis uses naturally occurring variation to estimate the causal effects of treatments, interventions, and risk factors on outcomes in the population from observational data. Under specific assumptions, instrumental variable methods can provide unbiased estimates of causal effects. This article explains these assumptions and the information and tests typically reported in instrumental variable studies, which can assess the credibility of the findings of instrumental variable studies.

In clinical practice, establishing causation is crucial for informed decision making in patient care. Instrumental variable analysis is increasingly used to provide evidence about causal effects in clinical research (see box 1 for glossary). Instruments are variables that are associated with the intervention, which have no uncontrolled common causes with the outcome and only affect the outcome via the intervention. They can be used to overcome measured and unmeasured confounding of intervention-outcome associations and provide unbiased estimates of the causal effects of an intervention on an outcome using observational data (fig 1). Instrumental variables are defined by three assumptions (box 2).

Box 1 **Glossary of terms used in instrumental variable studies**

**Concepts**

- Natural experiment: A source of variation in the likelihood of receiving an intervention in the real world that can be used to investigate the causal impact of an intervention.
- Instrumental variable: A specific variable in a dataset that is (1) associated with an intervention, (2) has no uncontrolled common causes with the outcome, and (3) only affects the outcome via the intervention.
- Fourth point identifying assumption: The assumption used to estimate the mean effect of the intervention on the outcome, without which it is only possible to estimate bounds for the effect of the intervention on the outcome.
- Local average treatment effect or complier average causal effect: The effect of an intervention on individuals whose intervention status is affected by the instrument.
- Counterfactual values: The patients’ outcomes had they been allocated to the other strategy (ie, the patients’ outcomes following intervention if they were assigned to control or the patients’ outcomes following control if they were assigned to intervention).

**Statistical methods**

- Reduced form: The instrument-outcome association, which, if the instrumental variable assumptions hold, is a valid test of the null hypothesis that the intervention does not affect the outcome.
- Wald estimator: The ratio of the instrument-outcome and instrument-intervention associations.^1^
- Two-stage least squares: An instrumental variable estimator. The first stage estimates the instrument(s)-intervention association(s) and uses these associations to predict the intervention values.^2^ The second stage uses the predicted interventions in a regression to estimate the effect of the intervention(s) on the outcome.

**Fig 1:**
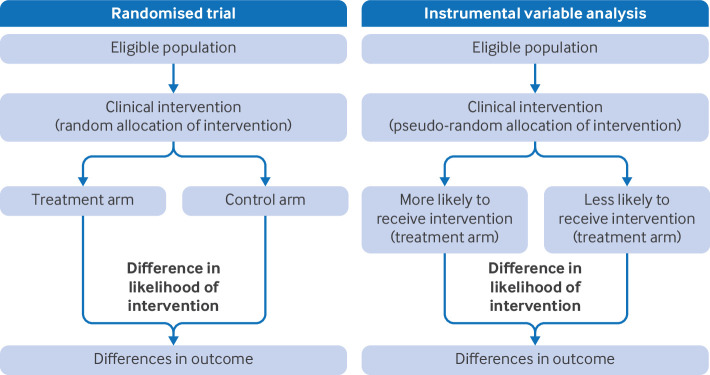
Similarities and differences between instrumental variable analysis and randomised controlled trials

Box 2 **Key assumptions that define instrument variables^3^**

- Relevance (IV1): The instrument must be associated with the intervention.
- Independence (IV2): The instrument and the outcome must have no uncontrolled common causes.
- The exclusion restriction (IV3): The instrument must only affect the outcome through the intervention.

Instrumental variable analysis has a long history (supplementary box 1), with applications in many fields, including healthcare and economics. The approach has increased in popularity owing to the availability of larger datasets, the recognition of the need to obtain reliable estimates when key covariates are not measured, and the use of different analytical assumptions.^4^ ^5^

Researchers increasingly use instrumental variable analyses to inform a wide range of clinical questions. For example, institutional variation in testing or treatment practices have been used as instrumental variables to estimate the effects of perioperatively testing for coronary heart disease on postoperative mortality rates,^6^ the relative safety of robotic versus laparoscopic surgery for cholecystectomy,^7^ and the length of storage of red blood cells and patient survival.^8^ Physicians’ preferences for treatments have been used to investigate the effects of cyclo-oxygenase-2 (COX-2) versus non-selective, non-steroidal anti-inflammatory drugs (NSAIDs) on gastric complications,^9^ ^10^ and the effects of conventional versus atypical antipsychotic drug treatments on mortality in elderly patients.^11^ Allocation to treatment in randomised controlled trials with non-compliance is an instrumental variable previously used to investigate the effects of flexible duty hour conditions for surgeons on patient outcomes and surgeons’ training and wellbeing^12^ and the effects of reducing amyloid levels on cognition.^13^ Distance from or time to admission to a particular type of hospital has been used as an instrument for receiving a specific treatment.^14^ ^15^

One of the most commonly used applications of instrumental variables is mendelian randomisation—using genetic variants as instrumental variables. The core principles of instrumental variable analysis still apply to mendelian randomisation and have been covered in detail and will not be discussed here.^16^ ^17^

Here, we provide a practical guide for researchers to read, interpret, and conduct instrumental variable studies using non-genetic observational data. In this article, we discuss why a study should use instruments, key concepts and assumptions, how to assess the validity of instrumental variable assumptions, and how to interpret results.

**Summary points**

Instrumental variable analysis is a research method that uses naturally occurring variation (ie, variation not controlled by researchers), such as policy decisions, clinical preferences, distance, or time, to provide evidence about the causal effects of interventions on outcomes from observational data
Instrumental variables can provide credible evidence about the causal effects even if other observational techniques have residual confounding, reverse causation, or other forms of bias
This article demonstrates how to perform an instrumental variable analysis using commonly available packages
In common with all empirical research methods, instrumental variable analysis depends on assumptions that readers and reviewers must assess
Many sources of evidence, using a range of assumptions, can help inform clinical decisions
A critical appraisal checklist is provided to help assess and interpret instrumental variable studies

## Clinical and public health implications

Researchers increasingly use large datasets of electronic medical records, registries, or administrative claims data to provide evidence about the effects of interventions on patient outcomes. An important limitation of these datasets is that while the large sample size allows for very precise results, they frequently have inadequate measures of critical confounders. Confounders are variables that affect the likelihood of receiving the intervention and that also affect the outcome (eg, previous neuropsychiatric diagnoses and the likelihood of being prescribed varenicline rather than nicotine replacement therapy for smoking cessation). Patients rarely receive interventions entirely randomly. Key confounders, such as morbidity and other indications for intervention, are often challenging or impossible to measure with sufficient accuracy from diagnosis or billing codes or are unmeasured or unmeasurable. Thus, matching individuals receiving the intervention with sufficiently comparable controls can be difficult or impossible. As a result, observational analysis of large scale databases could provide unreliable evidence about interventions’ comparative effectiveness and safety. This issue is challenging for clinicians and patients because they need reliable evidence of the causal effects of different interventions to make well informed decisions. Instrumental variables can provide an alternative source of evidence about the effects of different interventions and, while less precise than other approaches, might be less affected by individual level biases such as confounding by indication, where the indications for intervention also affect the likelihood of an outcome.

## Why use an instrument?

Most observational methods, such as multivariable adjusted regression or propensity score analysis, assume that it is possible to measure a sufficient set of confounders to account for all differences in the outcome between individuals given the intervention and control, except for those caused by the intervention.^18^ ^19^ However, the correct set of confounders is not always known, and even if they have been identified, measuring and accounting for baseline differences is extremely difficult, which can result in multivariable-adjusted and propensity score analyses having serious biases and providing misleading results. 

For example, COX-2 inhibitors were developed to cause fewer gastrointestinal complications than traditional NSAIDs and marketed to patients and physicians accordingly. As a result, patients prescribed these drug treatments typically were at higher risk of gastrointestinal complications at baseline. Thus, in observational datasets, patients prescribed COX-2 tended to have higher rates of gastrointestinal complications than patients prescribed NSAIDs, a difference that was not fully attenuated after adjustment for measured confounders. This result is because the pre-existing differences in the risk of gastrointestinal complications are very challenging to measure sufficiently, especially in electronic medical records, resulting in residual confounding by indication. 

Alternatively, patients prescribed nicotine replacement therapy for smoking cessation differ from those prescribed drugs such as varenicline: patients prescribed nicotine replacement therapy tend to be more unwell, be older, and have poorer mental health.^20^ However, electronic medical records or other datasets often do not record these differences. For example, patients might discuss smoking cessation with their general practitioner when they have preclinical symptoms of heart disease; these symptoms might not be perfectly captured in medical records.

Instrumental variable analysis offers an approach to deal with these problems. It relies on a distinct set of assumptions from other methods, which do not require measuring or knowing all the potential confounders of the intervention and outcome.

## What is an instrumental variable?

**Fig 2:**
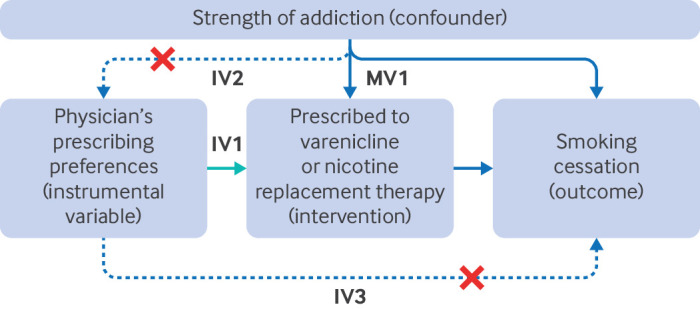
Assumptions of multivariable adjusted and instrumental variable studies. Physicians’ prescribing preferences are typically unmeasured; thus, normally, studies use prescriptions issued to the physicians’ previous patients to proxy for their preferences. Multivariable adjustment (MV1) assumes that a sufficient set of confounders can be measured to control for all open paths between the intervention and the outcome. In contrast, instrumental variable analysis assumes that there is an instrument that associates with the intervention (relevance; IV1), has no uncontrolled common cause with the outcome (independence; IV2), and only affects the outcome through the intervention (exclusion restriction; IV3)

The following three assumptions define instrumental variables. Firstly, the instrument is associated with the intervention of interest (relevance); secondly, it shares no uncontrolled common cause with the outcome (independence); and thirdly, it only affects the outcome through the intervention (exclusion restriction). Instruments only need to be associated with the likelihood of receiving the intervention; they do not necessarily need to cause it.^3^ Instrumental variable analyses exploit naturally occurring variation (the instrument) to estimate the impact of the intervention on an outcome. This variation can be due to clinical or policy decisions unrelated to unmeasured confounders. Box 2 defines these assumptions, and figure 2 uses a directed acyclic graph to represent these assumptions. Assessing the plausibility of the assumptions is critical to determining whether a proposed instrumental variable is valid and is discussed in detail below.

These assumptions can be defined unconditionally, or more often conditionally, on other important covariates in a dataset; for example, physicians’ prescribing preferences are usually conditioned on a patient’s age. If these assumptions are violated, for example, by residual confounding of the instrument-outcome association, then the results of an instrumental variable analysis can be more biased than other approaches, such as multivariable adjustment and propensity score. Thus, a key challenge for authors and readers of instrumental variable studies is determining whether the assumptions are plausible for the research question.

## Types of instruments

Numerous natural experiments have been proposed and assessed as potential instruments. These commonly include physician preference (eg, preference to prescribe one intervention versus another for a given diagnosis), access to intervention (eg, distance to a hospital with specific speciality staff or equipment), or randomisation (eg, in the context of a randomised controlled trial with non-compliance). Examples of these instruments are given below. Other sources of variation have also been used and are covered elsewhere.^21^

### Physician preference

Clinicians have preferences for many clinical decisions, such as testing, treatments, or diagnoses. These pre-existing preferences could be independent of the subsequent patients they see. For example, a physician might prefer prescribing nicotine replacement therapy over drug treatments such as varenicline.^20^ Studies generally cannot measure physicians’ preferences for one intervention or another, so they measure preferences in other ways. For instance, physicians’ prescribing preferences might be captured by looking at previous prescriptions for the interventions under consideration or, more rarely, surveys used to elicit preferences. Physicians’ prescriptions to their previous patients are often associated with the prescriptions they issue to their future patients. If this preference occurs in a way that is unrelated to the patient level confounders of their current patients, the independence assumption could hold. Physicians’ previously demonstrated preferences are consistently associated with their prescriptions to their current patients.^9^ ^10^ A potential weakness of physicians’ prescribing preferences as an instrument is that they might not be specific to the treatment of interest and could be associated with broader differences in care.

### Access

Access instruments include distance to hospitals,^14^ travel times to the hospital as a proxy for quicker treatment,^22^ the raising of the school leaving age as a proxy for education,^23^ and date of treatment as a proxy for choice of treatment.^24^ Here, for the instrumental variable assumptions to hold, access must associate with the likelihood of receiving the intervention but not directly affect the outcome or share any unmeasured confounders with the outcome. A potential weakness of studies using access based instruments is that geographical location and distance to healthcare facilities are often highly non-random and are related to important unmeasured confounders such as socioeconomic position.

### Random assignment in the presence of non-compliance

Treatment assignment in a randomised controlled trial with non-compliance or an encouragement design can be an instrumental variable.^25^ ^26^ ^27^ By design, random assignment should balance confounders between individuals assigned to the intervention and those assigned to the control. Conventional analyses of randomised trials report the intention-to-treat estimate, which is the difference in outcomes between participants assigned to the intervention and participants assigned to the control. However, if some trial participants do not comply with their allocation, the intention-to-treat estimate will underestimate the effects of taking the intervention because it will also reflect the effects of compliance. 

Instrumental variable analysis can be used to estimate the effects of taking the intervention, which can be estimated by assuming that the treatment assignment affects the likelihood of receiving the intervention in the same direction for all individuals (ie, the instrument has a monotonic effect if it increases the likelihood of the intervention for some individuals it does not decrease it for others). Under the monotonicity assumption, the instrumental variable estimate will reflect the complier or local average treatment effects (see box 3 for definitions). This parameter is the effect of the intervention on individuals whose treatment status was affected by the instrument. A limitation of random assignment is that assignment might alter behaviour in other ways, leading to violations of the exclusion restriction (eg, if individuals assigned to control in an unblinded trial seek treatment via other means). Examples of using allocation to treatment as an instrument include a cluster randomised trial of vitamin A supplementation with non-compliance.^25^ Treatment allocation can be used to estimate the effects of an underlying continuous risk factor, for example, the effects of reducing amyloid levels on cognition rather than the effect of being allocated to amyloid-lowering drug treatment.^13^ If the risk factor is continuous, then it is more challenging to interpret under monotonicity, and studies might make other assumptions (eg, assuming a constant effect of the risk factor).

Box 3 **Point identifying assumptions and interpretation**

The three core assumptions for instrumental variable analysis are only sufficient to estimate the bounds of a causal effect, which are the largest and smallest values consistent with the observed data. However, instrumental variable bounds are typically very wide, so most instrumental variable studies require a further fourth, point identifying assumption. Options for the fourth assumption include the constant treatment effect (IV4h), no effect modification (IV4n), no simultaneous heterogeneity (NOSH; IV4nosh), and monotonicity (IV4m).^2^ ^28^ ^29^

- The constant treatment effect assumption requires that the effect of the intervention on the outcome is the same for all individuals. For example, if the intervention of interest was an anti-hypertensive drug treatment such as angiotensin-converting enzyme (ACE) inhibitors, these inhibitors should give the same reduction in systolic blood pressure for all participants, regardless of any other characteristics.
- The no effect modification assumption requires that the intervention has the same effect on the outcome irrespective of the instrument’s value. For example, if the effects of ACE inhibitors are the same irrespective of physicians’ preference.
- The NOSH assumption requires that any heterogeneity in the effects of the instrument on the intervention is independent of heterogeneity in the effects of the intervention on the outcome. This assumption would hold if the variation in the effect of physician preferences on prescribing were not related to the treatment’s expected efficacy (ie, the instrument implicitly samples a representative sample of causal effects from the population).
- The monotonicity assumption requires that the effect of the instrument on the likelihood of receiving the intervention is always in the same direction (eg, the instrument only increases or decreases the likelihood of receiving the intervention). For example, a patient with a physician who prefers to prescribe ACE inhibitors will be more likely to receive an ACE inhibitor than a patient who attends a physician who prefers another anti-hypertensive drug.

**Assessment of point identifying assumptions**

These point identifying assumptions are untestable but falsifiable. The constant treatment effect assumption is potentially falsifiable by checking for differences in the implied effects of the intervention across covariates. For binary interventions with causal binary instruments and binary outcomes, monotonicity inequalities can falsify the monotonicity assumption.^30^ Cumulative distribution graphs for continuous interventions can assess this assumption.^2^ If the proposed instrument is a preference, assessing the plausibility of the monotonicity assumption is possible by conducting a preference survey.^31^ These surveys suggest that a strict definition of monotonicity is unlikely to be plausible, as there is substantial heterogeneity in clinical treatment decisions. However, Small and colleagues in 2017 proposed a more plausible assumption: stochastic monotonicity, which requires that the effect of the instrument on the exposure is monotonic conditional on a set of covariates.^32^

**Interpretation of instrumental variable estimates**

Instrumental variable estimates can be interpreted as the average treatment effect under the constant treatment effect, no effect modification, or NOSH assumptions. The constant treatment effect assumption identifies the average treatment effect by assuming the intervention has the same effect for all individuals. This assumption is most commonly used to identify the effects on continuous outcomes. However, this assumption can be implausible. For example, an intervention could only have a constant effect on a binary outcome if it entirely determined the outcome or did not affect it. In the example of ACE inhibitor use, it is implausible to assume that ACE inhibitors have the same effect on every individual in the population.

The no effect modification assumption identifies the intervention’s effect on those participants who receive the intervention by assuming that the effect of the intervention is independent of the instrument’s value. For example, in a randomised controlled trial with an encouragement design where the intervention is an encouragement to take a treatment, allocation to the intervention or control arm does not change the effect of the treatment. This assumption can identify interventions’ effects on binary outcomes and estimate causal risk and odds ratios. Finally, the NOSH assumption requires that heterogeneity in the effects of the instrument on the likelihood of receiving the intervention must be independent of heterogeneity in the effect of the intervention on the outcome to be interpreted as the average treatment effect.^29^

Instrumental variable estimates can be interpreted as reflecting a local average treatment effect using the monotonicity assumption. The monotonicity assumption identifies the effects of the intervention on those individuals whose intervention status was affected by the instrument. This assumption is typically, but not exclusively, applied to binary instruments and interventions.^33^ Individuals who either always take the intervention or never take the intervention, regardless of whether they were assigned to it, will not be affected by the instrument. Two groups of individuals remain: those who only take the intervention when they are assigned to it (known as compliers), and those who only take the intervention when they are not assigned to it (known as defiers). The monotonicity assumption assumes that there are no defiers in the sample. For example, physicians’ prescribing preferences could have a monotonic effect if patients prescribed nicotine replacement therapy who attended a physician who previously prescribed varenicline would also have been prescribed nicotine replacement therapy by a physician who previously prescribed nicotine replacement therapy (and vice versa).

The instrumental variable assumptions need to be assessed and considered for each application (box 4). Just because the assumptions are plausible for one treatment or population does not mean that they will be valid in another.

Box 4 **Critical appraisal checklist for evaluating instrumental variable studies**

Readers of instrumental variable studies could consider the following questions:

**Core instrumental variable assumptions**

- Is there evidence that the instruments are associated with the intervention of interest? Does the study report a first stage partial F statistic?
- Are the instruments associated with measured potential confounders of the intervention and outcome?
- Are there likely to be different confounders of the instrument-outcome association than the intervention-outcome association?
- Is the proposed instrument likely to affect the outcome via mechanisms other than the intervention of interest?
- Do the authors use negative control outcomes to investigate the plausibility of the instrumental variable assumptions?

**Fourth instrumental variable assumption**

- Do the authors report the fourth instrumental variable assumption?
- Do the authors describe their estimand and how it relates to clinical practice?

**Methods**

- Does the study clearly state the instrumental variable estimator used in the analysis?
- For two-stage least squares, are the same covariates included in both stages of the analysis?

**Data presentation**

- Do the authors present the instrument-outcome association, an instrumental variable estimate, or both?
- If they provide an instrumental variable estimate, do they compare it with the multivariable-adjusted estimate?
- Was the definition of the instrument prespecified, or was the definition of the instrument chosen based on the data under analysis?
- Do the authors provide the code they used to allow researchers to reproduce their findings?

**Interpretation**

- If the instrumental variable estimate is similar to the multivariable adjusted estimate and provides evidence consistent with a causal effect, could it be due to weak instrument bias in a single study or confounding of the instrument-outcome association?
- If the instrumental variable estimate differs from the multivariable adjusted estimate and provides little evidence of a causal effect, could this be due to weak instrument bias or confounding?
- Are the 95% confidence intervals of the estimate sufficiently precise to test for differences with the multivariable adjusted estimate and detect a clinically meaningful difference?

**Clinical implications**

- Do the results triangulate with other forms of evidence?
- If a randomised clinical trial is not feasible or unlikely to be conducted in the short term, and there is existing evidence from multiple instrumental variable studies, and other robust study designs converge on consistent results, this information may help guide patient care; for example, informing clinical guidelines or regulatory decisions.

## Assessment of instrumental variable assumptions

Directed acyclic graphs provide a convenient and transparent way to depict and explain the assumptions required for an applied instrumental variable analysis.^34^ ^35^ ^36^ Researchers can adapt the structure used in figure 2 for specific research questions. Studies can then use empirical data to assess whether the three core assumptions for instrumental variables hold.

The first instrumental variable assumption (relevance) states that the instrument must be strongly associated with the likelihood of taking the intervention. The strength of the instrument-intervention association is easily testable. For example, in the study of drug treatments for smoking cessation, we found that physicians who had previously prescribed varenicline were 24 percentage points (95% confidence interval 23 to 25) more likely to prescribe varenicline to their subsequent patients than physicians who had previously prescribed nicotine replacement therapy. However, a difference in treatment rates across instrument values is insufficient to measure instrument strength because it does not reflect the sample size. In a small study of a few hundred patients, even a very large difference in treatment rates across the instrument’s value will provide very little information about the effects of treatment.

In contrast, the first stage partial F statistic of the regression of the intervention on the instrument indicates both the strength of the association and the total sample size. The first stage partial F statistic in an instrumental variable analysis is analogous to the sample size in a randomised controlled trial. Most instrumental variable estimation packages in Stata and R (such as ivreg2 or AER, respectively)^37^ ^38^ will report this F statistic by default. A value above 10 is considered strong and unlikely to lead to weak instrument bias.^39^ However, an F statistic above 10 does not guarantee that an instrumental variable study will have sufficient statistical power to detect an effect size of interest.

The remaining assumptions are untestable, so they cannot be proven to hold, but they are falsifiable.^31^ ^40^ An assumption is falsifiable if it is possible to use empirical data to disprove it. The independence assumption can be falsified by testing the instrument-covariate associations using covariate balance and bias component plots,^41^ ^42^ or randomisation tests.^43^ If the instrumental variable assumptions hold, no associations between the instrument and alternative pathways or other covariates that predict the outcome should be detected.^44^ The exclusion restriction is falsifiable by demonstrating that other variables are affected by the instrument, which also affects the outcome. For example, in a study of angiotensin-converting enzyme (ACE) inhibitors for cardiovascular disease, if physicians who are more likely to prescribe these inhibitors are also more likely to prescribe statins, which also affect cardiovascular disease, the exclusion restriction assumption would be violated. 

Another way to falsify the independence and exclusion restriction assumptions is using negative controls to investigate whether the instrument predicts the outcome in subgroups of the population for which the instrument does not affect the likelihood of receiving the intervention. If evidence indicates that the instrument affects the outcome, even in subgroups where the instrument does not affect the likelihood of receiving the intervention, the instrumental variable assumptions are unlikely to be plausible (eg, by using patients who do not have hypertension (eg, children) who were treated for other indications by physicians who preferred ACE inhibitors). Falsification tests are useful indicators of how plausible the assumptions are likely to be, however, and failure to falsify an assumption does not prove it holds. For example, if the instrument was associated with an unmeasured confounder of the intervention and the outcome, this association would not be evident in a covariate plot that only included measured covariates.

A further way to assess the plausibility of assumptions is to investigate any differences (heterogeneity) in the effect sizes implied by different instruments. This approach requires more than one instrument (which, when there are more instruments than interventions, is technically known as being over-identified). If more than one instrument is affecting the likelihood of receiving the intervention (eg, physicians’ preferences and distance to the healthcare facility), the heterogeneity in the effects of the intervention implied by each instrument could indicate violations of the instrumental variable assumptions. Bonet’s instrumental variable inequality tests can also falsify binary interventions’ exclusion restriction and independence assumptions.^45^

## How to generate instrumental variable estimates

Instrumental variables can test whether an intervention affects an outcome and estimate the magnitude of that effect. The simplest estimator is the instrument-outcome association (reduced form; box 1), which can be estimated using regression methods (eg, linear or logistic regression methods). This estimator does not estimate the magnitude of the effect of the intervention on the outcome. However, under the instrumental variable assumptions, it is a valid test of the null hypothesis that the intervention does not affect the outcome. An advantage of this test is that it is simple, requires the fewest and weakest assumptions, and can test for the existence of an effect. A disadvantage is that it does not provide a scale for the effect of the intervention on the outcome, limiting the interpretation of the results. Ideally, we want to know the average effect of the intervention (also known as the average treatment effect), and not just the effect of the instrument. For example, researchers and readers might be more interested in the effect of prescribing varenicline or nicotine replacement therapy (the intervention) on their current patient than the effect of physicians’ previous prescriptions for smoking cessation treatment (the instrument) on smoking cessation rates (the outcome).

Several instrumental variable estimators can estimate the average treatment effect. Some of the most used instrumental variable estimators are covered below. However, these methods were largely developed to estimate average treatment effects for normally distributed instruments, exposures, and outcomes assuming linear mechanisms. Although, in practice, these methods are widely used for binary outcomes or non-linear mechanisms (sometimes with the same or a different name), the interpretation can be difficult and more advanced methods might be required. 

If only one instrument is available, then the average effect of the intervention on an outcome can be estimated using instrumental variable estimators, such as the Wald estimator, which is the ratio of the instrument-outcome association divided by the instrument-intervention association. This estimator rescales the instrument-outcome association to the intervention scale and indicates the effect of a unit change in the intervention on the outcome. For example, if patients prescribed smoking cessation treatments by physicians who previously prescribed varenicline were 1 percentage point more likely to cease smoking (the instrument-outcome association) and 10 percentage points more likely to be prescribed varenicline (the instrument-intervention association), then the Wald estimate would be −0.01 ÷ 0.1 = −0.1. This estimate would imply that prescribing varenicline increases the absolute probability of stopping smoking by 10 percentage points.

When a study has one or more instruments available, for example, if a study used physicians’ preferences and distance to healthcare facility as instruments, then the effects of the intervention on the outcome can be estimated using a two-stage least squares estimator. This estimator comprises two regressions or stages. The first stage is a regression of the intervention on the instruments, which can predict the intervention value based on the instrument values. The second stage is a regression of the predicted intervention status on the outcome. The estimated coefficient on the predicted value is the instrumental variable estimate of the effect of the intervention on the outcome. A simulated example and the formulas are provided in the supplementary materials. It is usually essential that both stages of instrumental variable analysis contain the same covariates.^46^ However, this approach will not account for the estimation error from the first stage and is likely to give incorrect standard errors and confidence intervals. Typically, most analyses use a package such as ivreg2 in Stata or AER or ivreg packages in R,^37^ ^38^ which compute the instrumental variable estimates in one step and integrates the estimation errors from both stages.

Different types of outcomes require different instrumental variable estimators, which rely on logic similar to the two-stage least squares estimator. Commonly used estimators include:

- Continuous outcomes: Mean differences (eg, effects of smoking cessation treatment on body mass index using physicians’ prescribing preferences^20^) can be estimated using additive structural mean models.^2^
- Binary outcomes: Causal risk differences, odds ratios, and risk ratios (eg, estimating the effects of coronary bypass surgery on mortality^14^) can be estimated using additive, logistical, and multiplicative structural mean models and control function approaches.^33^ ^47^ ^48^
- Survival outcomes: Methods using instrumental variables with survival outcomes, which adopt a similar approach to two-stage least squares, or the control function approach,^49^ have been developed to allow for covariate and outcome dependent censoring.^50^ For example, estimating the effects of screening frequency on colorectal cancer diagnoses using international differences in screening policies.^51^
- Instrumental variable quantile regression: Non-linear effects of the intervention can be estimated using instrumental variable quantile regression.^52^ ^53^ ^54^ For example, investigating whether the effects of a unit increase in body mass index on healthcare costs differ between underweight and overweight individuals.^55^

Methods for instrumental variable estimation is an area of active methodological development, spanning statistics, econometrics, and computer science. Examples include estimators combining instrumental variable analysis and matching^56^ and estimators using machine learning.^57^ ^58^ ^59^

## Data for instrumental variable studies

Instrumental variable studies typically require measures of the instrument, the intervention, and the outcome for individual level data analysis using the same sample of people. This straightforward approach allows the most flexibility to test and evaluate the instrumental variable assumptions. However, integrating additional external datasets can improve the power and precision of instrumental variable analyses using an approach known as two-sample instrumental variable analysis.^60^ This approach estimates the instrument-intervention association in one sample and the instrument-outcome association in another, from which the Wald estimator can be calculated. For example, a study could estimate the effects of policy reform on educational attainment using census data from the entire population but estimate the effects on health outcomes in a cohort study subsampled from the same underlying population.^61^ A two-sample instrumental variable analysis does not need measures of the intervention or the outcome in all samples, which can increase power considerably, particularly when the outcome is rare or difficult to measure.

## Summary

Instrumental variable analysis can provide reliable evidence about the causal effects of an intervention, even if the intervention-outcome association is affected by unmeasured confounding. Key to conducting and reading instrumental variable studies is assessing the plausibility of the three core assumptions on instrumental variables. Does the instrument strongly associate with the intervention? Is there a rationale for why the instrument-outcome association is less likely to have confounding than the intervention-outcome association? Is there evidence that measured covariates are less strongly associated with the instrument than the intervention? Are alternative pathways available that could mediate the effects of the instrument?

Instrumental variable analysis can provide a valuable complement to other forms of observational analysis. Unlike other approaches, instrumental variables have distinct assumptions and can strengthen inferences when combined with other sources of evidence. The increasing amount of data available for clinical research means that there is a growing opportunity to use these methods to improve patient care.

## Web extra

Extra material supplied by authors

Web appendix: Supplementary materials

## Notes

Provenance and peer review: Not commissioned; externally peer reviewed.
