# Supplementary material for: Reading and conducting instrumental variable studies: guide, glossary, and checklist
Source: BMJ. 2024 Oct 14;387:e078093. doi: 10.1136/bmj-2023-078093 (PMC11472180; doi:10.1136/bmj-2023-078093)
Supplement: Supplementary file 1 — Web appendix: Supplementary materials [file walv078093.ww.pdf]

Reading and conducting instrumental variable studies: a guide, glossary and checklist for clinicians

Venexia Walker research fellow,[1,2] Eleanor Sanderson Lecturer in Medical Statistics[1,2], Michael G Levin cardiologist[3,4], Scott Damrauer William Maul Measey Associate Professor of Surgery[3,4], Timothy Feeney Research editor[6], and Neil M Davies professor of Medical Statistics[7,8,9]

Supplement

Supplementary Box 1: A Brief History of Instrumental Variables

Instrumental variable (IV) analysis has a rich history in econometrics and has expanded to various fields, including medicine and social sciences. This statistical technique emerged as a powerful tool for addressing endogeneity and establishing causal relationships. Sewall Wright and Philip Wright originally proposed instrumental variables in 1926[66]. Here are some key examples:

Angrist and Krueger (1991): In their influential study, Angrist and Krueger used changes in compulsory schooling laws as an instrumental variable to estimate the causal effect of education on labour market outcomes, such as earnings[67]. This work demonstrated the power of instrumental variables in social sciences and economics.

Newhouse and McClellan (1993): Newhouse and McClellan conducted a seminal study using geographic variation in healthcare spending as an instrumental variable to examine the causal impact of healthcare expenditures on patient outcomes[12]. This study shed light on the effectiveness and efficiency of healthcare spending, contributing to health policy discussions.

Brookhart et al. (2006): A significant contribution to pharmacoepidemiology, Brookhart et al. used prescribing preferences of physicians as an instrumental variable to assess the causal effects of medications on patient outcomes[7]. This study highlighted the applicability of instrumental variable analysis in addressing confounding bias in observational studies.

Hernán and Robins (2006): Hernán and Robins provided an exposition of the assumptions required for instrumental variable analysis[1]. They highlighted the approach's potential value in epidemiological and clinical research, particularly in the absence of randomised controlled trials.

Supplementary Box 2: Instrumental variable estimation

The two most common instrumental variable estimators are the Wald estimator and the 'two-stage least squares' (2SLS) estimator. The Wald estimator can only be used with a single instrument. In contrast, two-stage least squares can use multiple instruments.

With a binary instrument (Z) and binary or continuous intervention (X) and outcome (Y) the Wald estimator is:

$$\widehat{\beta_{IV}} = \frac{E(Y|Z=1) - E(Y|Z=0)}{E(X|Z=1) - E(X|Z=0)} \quad \text{(i)}$$

This is the difference between the outcome when the instrument takes the value 1 and when the instrument takes the value 0 divided by the difference in the intervention when the instrument takes the value 1 and the value 0.

If the instrument is continuous, the Wald estimator is the ratio of the instrument-outcome ($\widehat{\beta_{ZY}}$) and instrument-intervention ($\widehat{\beta_{ZX}}$) associations estimated using linear regression.

$$\widehat{\beta_{IV}} = \frac{\widehat{\beta_{ZY}}}{\widehat{\beta_{ZX}}} \quad \text{(ii)}$$

Where β_{ZY} is obtained from the estimation of the regression:

$$Y = \alpha_Y + \beta_{ZY} Z + u_Y \quad \text{(iii)}$$

Where Y is a vector containing the outcome for each individual, Z is a vector containing the instrument for each individual and u_Y is a random error term. And β_{ZX} is obtained from the estimation of the regression

$$X = \alpha_X + \beta_{ZX} Z + u_X \tag{iv}$$

Where X is a vector containing the outcome for each individual, Z is a vector containing the instrument for each individual and u_X is a random error term. The two-stage least squares estimator can be estimated by predicting the value of the intervention using the observed values of the instrument. This is obtained by estimating equation (iv) and predicting X, indicated X from the estimated values of $\hat{\alpha}_X$and $\hat{\beta}_{ZX}$.

$$\hat{X} = \hat{\alpha}_X + \hat{\beta}_{ZX} Z \tag{v}$$

The instrumental variable estimate of the effect of receiving the intervention is then obtained by replacing the intervention with the predicted intervention status in the 'second stage' regression of the intervention on the outcome.

$$Y = \alpha_{Y_{iv}} + \beta_{IV} \hat{X} + u_{Y_{iv}} \tag{vi}$$

This second stage regression can be estimated using linear regression for continuous outcomes or logistic regression for a binary outcome. In each case, the standard errors must account for the uncertainty in the prediction of intervention X in the second stage. This correction is done automatically in instrumental variable regression packages such as ivreg2 in Stata or ivreg in the AER R package.

Supplementary Box 3: Instrumental variable analysis of COX-2s vs traditional NSAIDs using physicians' prescribing preferences

This example uses a simulated random sample from the population. It simulates a study investigating the effects of two types of anti-inflammatory drugs, traditional NSAIDs (e.g. ibuprofen) vs. COX-2 selective inhibitors (COX-2s, e.g. celecoxib). The dataset contains data from 100,000 patients, and it is a patient-level file, i.e. each patient has a single row. In the dataset, the intervention is indicated by the variable 'prescribed_cox_2'. It equals one if the patient had a COX-2 and zero if they had a traditional NSAID. The outcome of interest is whether the patient subsequently had a gastrointestinal complication (variable 'has_gi_event') equal to one or did not have a complication when the outcome is equal to zero. The dataset is called iv_example.csv. There are 100,000 observations with variables on treatment, physician who prescribed the treatment, age, and sex. We will use the information on the physician who prescribed the treatment to create an instrument and estimate the effects of prescribing COX-2s versus traditional NSAIDs.

1. **Create the instrument:** the physician's previous prescription. The variable visit order indicates the order in which the patients visited their GP. Create a variable equal to one if the physician previously prescribed a COX-2 and equal to zero if they previously prescribed a traditional NSAID.
2. **Test the relevance assumption (IV1)**. Are the physicians' previous prescriptions associated with their subsequent prescriptions?

Supplementary Table 1: Association of instrument and likelihood of treatment

		Confidence intervals			
	**Risk difference per 100**	**Lower**	**Upper**	**R^2**	**F-statistic**
Prior Rx Cox-2	10.7	10.1	11.4	1.1%	1156

R^2 is the proportion of variability explained in the outcome variable of a regression by the covariates. The R^2 value for the prior prescription instrument is 1.1%. Since the R^2 statistic is small, we know that the resulting IV estimates will be imprecise and have wide confidence intervals.

The F statistics for the (first stage) regression of prescribed COX-2 on prior prescription is 1156. Economists often refer to an instrument with a first-stage F statistic less than 10 as a "weak instrument", i.e. an instrument which will give an IV estimate with a relatively large finite sample bias. However, it is essential not to just select instruments, or search through different definitions of the instrument with F statistics greater than 10 in the dataset under analysis as this can lead to bias via winner's curse.

The linear probability model estimates the association between the instrument and the likelihood of receiving the intervention on the absolute probability scale, i.e. risk differences.

3. **Evaluate the independence assumption**: Investigate the plausibility of the third instrumental variable assumption, independence. Do the instruments associate with the measured confounders?

Supplementary Table 2: Association of instrument, prior prescription and measured confounders

Outcome:		**Confidence intervals**		
	**Mean/Risk difference per 100**	**Lower**	**Upper**	**p-value**
Age	-6.1	-12.3	0.00	0.05
Female	-0.4	-1.0	0.2	0.22

There was little evidence of association between any of the instruments and age or sex. Of course, we cannot check for associations with unmeasured confounders. The simulated dataset includes unmeasured confounders.

4. **Estimate multivariable-adjusted regression**: Are prescriptions of COX-2s associated with a higher or lower risk of gastrointestinal events? What happens when you adjust for the observed covariates?

Supplementary Table 3: Association of prescriptions of COX-2s and gastrointestinal complications, unadjusted and adjusted for age and sex

Outcome:		**Confidence intervals**		
	**Risk difference per 100**	**Lower**	**Upper**	**p-value**
Unadjusted	5.1	4.9	5.4	<0.001
Adjusted	0.2	0.0	0.5	0.14

The unadjusted linear regression estimates show that patients prescribed COX-2s are 5.1 (95% CI: 4.9, 5.4) percentage points more likely to have a gastrointestinal adverse event compared to those prescribed traditional NSAIDs. This observational estimate attenuates after adjustment for age and sex to 0.2 (95% CI: 0.0, 0.5). The true effect of COX-2s on the gastrointestinal events was simulated in this dataset as -8.3 per 100 patients treated (i.e. fewer events in those prescribed COX-2s), so we can see that this analysis is still biased by an unmeasured confounder.

4. **Estimate the Wald estimator:** The instrumental variable ratio estimator (Wald type) is the instrument outcome association divided by the instrument-intervention association.

The instrument-outcome association is -0.006. The instrument-exposure association is 0.11. Putting these values into the Wald estimator results in the following estimate = -

0.006/0.11=-0.06, or 6.0 fewer events per 100 patients treated. Since this dataset was simulated under the instrumental variable assumptions, this IV estimate is close to the true effect of -8. Of course, it is not possible to know the “true” effect in real datasets.

6. Estimate two-stage least squares estimator: again using the prior prescription as the instrumental variable. Compare this with the ratio estimate above.

Supplementary Table 4: Two-stage least squares estimates of the effect of COX-2s on gastrointestinal complications, unadjusted and adjusted for age and sex

Outcome:		**Confidence intervals**		
	**Risk difference per 100**	**Lower**	**Upper**	**p-value**
2SLS	-6.0	-8.7	-3.3	<0.001
2SLS 3 prior Rx	-6.6	-8.3	-4.9	<0.001

We can see that with a single instrument, two-stage least squares gives the same estimate as the ratio estimator. We can increase the precision of the instrumental variable estimator by including multiple instruments, in this case, prior prescriptions.

7. Specification tests. Investigate the endogeneity test and overidentification test using -ivregress- postestimation commands.

We can test whether there is any evidence of differences between the multivariable-adjusted and instrumental variable results using an endogeneity test, which gives a chi^2(1)=198, p-value<0.001. This test rejects the null hypothesis that there are no differences between the two estimates. One explanation is if the multivariable-adjusted regression suffers from residual confounding.

Because we have multiple instruments, we can use the over-identification tests to investigate if there is any heterogeneity in the effects implied by each instrument. Here, we have three instruments, and the test gives chi^2(2)=2.2, p-value=0.34. This test means we

cannot reject the null hypothesis that the effects of COX-2s on gastrointestinal complications implied by each of the three instruments are the same.
